# Optical Biosensor for the Detection of Infectious Diseases Using the Copolymer F8T2 with Application to COVID-19

**DOI:** 10.3390/s22155673

**Published:** 2022-07-29

**Authors:** Maiara de Jesus Bassi, Maritza Araujo Todo Bom, Maria Luisa Terribile Budel, Emanuel Maltempi de Souza, Marcelo Müller dos Santos, Lucimara Stolz Roman

**Affiliations:** 1 Physics Department, Federal University of Paraná, Curitiba 81531-980, Brazil; lsroman@fisica.ufpr.br; 2 Biochemistry Department, Federal University of Paraná, Curitiba 81531-980, Brazil; maritzabom@gmail.com (M.A.T.B.); malu.budel@ufpr.br (M.L.T.B.); souzaem@ufpr.br (E.M.d.S.); marcelomuller@ufpr.br (M.M.d.S.)

**Keywords:** biosensor, F8T2, photoluminescence, anti-RBD/RBD binding

## Abstract

The coronavirus pandemic caused by severe acute respiratory syndrome coronavirus 2 (SARS-CoV-2) has accelerated the development of biosensors based on new materials and techniques. Here, we present our effort to develop a fast and affordable optical biosensor using photoluminescence spectroscopy for anti-SARS-CoV-2 antibody detection. The biosensor was fabricated with a thin layer of the semiconductor polymer Poly[(9,9-di-n-octylfluorenyl-2,7-diyl)-alt-2,2′-bithiophene-5,5′-diyl)] (F8T2) as a signal transducer material. We mounted the biosensors by depositing a layer of F8T2 and an engineered version of RBD from the SARS-CoV-2 spike protein with a tag to promote hydrophobic interaction between the protein and the polymeric surface. We validated the biosensor sensitivity with decreasing anti-RBD polyclonal IgG concentrations and challenged the biosensor specificity with human serum samples from both COVID-19 negative and positive individuals. The antibody binding to the immobilized antigen shifted the F8T2 photoluminescence spectrum even at the low concentration of 0.0125 µg/mL. A volume as small as one drop of serum (100 µL) was sufficient to distinguish a positive from a negative sample without requiring multiple washing steps and secondary antibody reactions.

## 1. Introduction

The current infectious disease COVID-19, which is caused by a coronavirus and which causes severe acute respiratory syndrome type 2 (SARS-CoV-2), has caused a serious, global pandemic in recent years. The first known cases emerged in the end of 2019 in China, and by the first half of 2020, the disease had already reached all continents and had killed thousands of people every day.

In order to face outbreaks such as the COVID-19 pandemic, the development of new biosensors capable of detecting viral infectious diseases in a fast, accurate, and accessible way has been decisive [1]. Here, we highlight a microfluidic immunosensor for fast and high-sensitivity measurements of the SARS-CoV-2 N protein in serum [2] and an ensemble machine-learning-based COVID-19 detection system, aiming to aid clinicians in diagnosing this virus effectively [3].

Biosensors are often described as three-element systems, consisting of a bioreceptor (antigen, antibody, enzyme, DNA/RNA), a transducer, and a signal-processing unit. A transducer converts a biochemical signal, resulting from the interaction of a biological component, into a measurable signal. Thus, when the interaction between the analyte and the bioreceptor occurs, a quantifiable signal is generated, which can be optical, electrochemical, thermometric, piezoelectric, magnetic, or micromechanical [4,5,6].

Optical biosensors have received considerable interest in the detection of pathogens due to their sensitivity and selectivity [7,8]. The optical signal is produced by specific biological interactions with the analyte, as in other detection technologies, but with the advantage of not requiring a direct electrical connection. Different optical properties, such as absorption, fluorescence, internal reflection, and surface plasmon resonance, can be explored to monitor biorecognition in biosensors [9,10,11,12].

Alternative materials have been studied for the development of new biosensors [13,14]. Here, we will highlight the conjugated polymers, which are excellent platforms for immobilizing biomolecules, since they are known to provide better signal transduction, presenting excellent sensitivity, selectivity, durability, and flexibility [15,16,17]. Those characteristics facilitate the transfer of electrons from biochemical reactions, which is fundamental for the development of biosensors [18,19,20]. Furthermore, the application of conjugated polymer inks in biosensors is interesting since they are compatible with the roll-to-roll processing used in creating electronic devices on a roll of flexible plastic substrates.

Several conjugated polymers have been used in this area, such as PPy for the impedimetric detection of SARS-CoV-2 at various stages of viral infection [21]. The superhydrophobic PTFE can be used in lab-on-a-chip and multisensor devices, as well as in biological cults [17]. PTTzFr has been used for glucose detection [15], and PFBT has been used for hydroquinone detection [16].

In this study, we report the development of an optical immunosensor based on antigen–antibody binding to diagnose the COVID-19 infectious disease with the great advantage of not using electrodes. For this development, we used the Poly[(9,9-di-n-octylfluorenyl-2,7-diyl)-alt-2,2′-bithiophene-5,5′-diyl)] (F8T2) copolymer as a signal transducer material. Although F8T2 has a wide application in organic electronics [22,23,24,25,26,27], its use in the development of optical biosensors makes this study quite innovative.

Studies were performed using the RBD (receptor-binding domain) antigen from the SARS-CoV-2 spike protein, engineered to firmly bind to the F8T2 surface, purified IgG anti-RBD antibody produced in rabbits, and finally, in place of the anti-RBD, human serum from patients who presented both positive and negative results in a real-time reverse transcription polymerase chain reaction (RT-PCR) exam for COVID-19. The identification of viral genetic material by RT-PCR is the gold standard for COVID-19 diagnosis, as it identifies the RNA from SARS-CoV-2 in nasal/oral swabs. The RBD is a binding domain within the spike S1 subunit that recognizes the human receptor ACE2 (angiotensin-converting enzyme 2), allowing the virus to enter host cells [28].

## 2. Materials and Methods

### 2.1. Materials

For this study the following materials were used: copolymer F8T2 (purchased from Lumtec, New Taipei City, Taiwan) solubilized in o-dichlorobenzene in a concentration of 4 mg/mL; 100 µL of recombinant RBD antigen of the SARS-CoV spike protein produced in the bacteria *Escherichia coli*, 100 µL of bovine serum albumin (BSA) surface blocker at a concentration of 2%; 100 µL of anti-RBD IgG polyclonal antibody of the SARS-CoV-2 spike protein produced in rabbits (purchased from Sino Biological^®^, Beijing, China) at a concentration of 1 µg/mL, and 100 µL of human serum from patients who showed both positive and negative results on the RT-PCR test for COVID-19. In this study, human serum was diluted 1000 and 3000 times. With the exception of F8T2, all other materials were deposited by dripping solution and solubilized in 50 mM Tris-HCl buffer, 150 mM NaCl.

### 2.2. Over-Expression and Purification of Recombinant SARS-CoV-2 Spike RBD

*E. coli* BL21(λDE3) carrying the pET6HisRBD-SBD plasmid was cultivated in 100 mL of LB in 500 mL Erlenmeyer flasks at 30 °C for 24 h. The culture was divided into 10 tubes of 10 mL each and centrifuged at 5000× *g* and 4 °C for 10 min. The supernatant was discarded, and the pellet resuspended in Buffer A (Tris-HCl 50 mM pH 8.0, 150 mM NaCl), transferred to 1.5 mL tubes, and sonicated 5 times (10 s of sonication, 10 s of resting) in an ice bath. After centrifugation (12,000× *g*, 4 °C, 10 min), the soluble fraction was discarded, and the inclusion bodies (IBs) washed 3 times with 1 mL of Buffer B (Tris-HCl 50 mM pH 8.0, 0.5% Triton X-100, 1 M urea). The washed IBs were dissolved with 1 mL of 8 M (mol/L) urea at room temperature, with pipetting up and down 10 times. The solution was not wholly translucent after solubilization with urea; however, there was enough soluble RBD-SBD to move to the refolding step. A quantity of 1 mL of denatured protein solution was transferred to a 12 kDa-cutoff dialysis tubing cellulose membrane and dialyzed against 1 L of Buffer C (50 mM Tris-HCl, pH 9.0, 150 mM NaCl) for 24 h at room temperature. The dialyzed solution was centrifuged (12,000× *g*, 4 °C, 10 min) and the supernatant injected at a flow rate of 1 mL/min in a 1 mL HiTrap Chelating charged with 100 mM NiCl_2_ and equilibrated with Buffer D (50 mM Tris-HCl, pH 9.0, 150 mM NaCl, 5% glycerol, 20 mM imidazole). The column was washed with 12 column volumes of Buffer D. The RBD-SBD was eluted in Buffer E (50 mM Tris-HCl, pH 9.0, 150 mM NaCl, 200 mM imidazole). The remaining imidazole was removed, dialyzing the protein against 2 L of Buffer C for 24 h at room temperature. 

### 2.3. Construction of the Optical Biosensor

After the F8T2 had been solubilized, it was deposited by spin coating on a previously cleaned glass substrate and submitted to thermal treatment at 100 °C. In order to detect IgG antibodies against the SARS-CoV-2 virus, 3 structures for biosensor formation were tested. In the first test, the biosensor was manufactured using the GLASS/F8T2/RBD/anti-RBD configuration. For the second test, the BSA surface blocker was used between the RBD and the anti-RBD layers. Finally, in the third test, the anti-RBD was replaced with COVID-19 positive and negative blood sera. Each layer was dried under ambient temperature and pressure. Before each deposition, the films were immersed in distilled water and allowed to dry at room temperature and pressure, ensuring the elimination of the solvents used.

### 2.4. Measurement

In this study, UV–Vis spectra were obtained on a Shimadzu spectrophotometer, model NIR 2101, and photoluminescence measurements were performed using the Fluorolog^®^-3 spectrofluorometer using an excitation at 450 nm.

## 3. Results and Discussion

In this study, three different optical biosensor configurations were developed to assess the detection of IgG antibodies against the SARS-CoV-2 virus.

**Figure 1:**
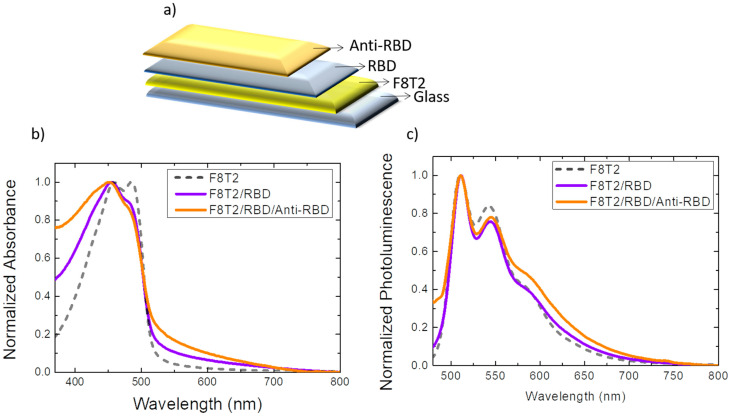
(**a**) Assembly diagram of the optical biosensor configuration GLASS/F8T2/RBD/anti-RBD. Optical properties of films for the optical biosensor construction: (**b**) normalized absorption spectrum; (**c**) normalized photoluminescence spectrum (excitation at 450 nm). Absorption and photoluminescence spectra of the F8T2 film were plotted by comparison.

In the first configuration, shown in Figure 1a, the optical biosensor was produced using F8T2 copolymer as the primary surface. This material was used as a physical–chemical signal transducer. Then, the RBD antigen and the anti-RBD antibody were deposited. Anti-RBD was used as the target analyte, that is, the recognition element of interest.

Figure 1b,c shows the absorption and photoluminescence spectra, respectively, after the deposition of each layer, until the formation of the biosensor was complete. As a reference for this analysis, the absorption spectrum of F8T2 copolymer has two defined maxima in the region of 460 nm and 486 nm. With the immobilization of the RBD antigen on the F8T2 surface, together with the anti-RBD target analyte, it was possible to observe that the absorption spectrum was smooth, with a maximum peak at 450 nm. A subtle amplification in the spectrum was also observed in the region from 370 nm to 450 nm and in the region from 515 nm to 635 nm, where the highest magnification refers to the film with all the layers of the biosensor.

Compared to absorption, the F8T2 photoluminescence spectrum also features 2 maximum peaks at 510 nm and 543 nm and a more discrete peak in the 585 nm region. With the immobilization of RBD antigen followed by anti-RBD, the peak intensity in the region of 543 nm was lower, and the film showing all the layers of the biosensor exhibited a small variation in intensity in the region of 570 nm–650 nm.

**Figure 2:**
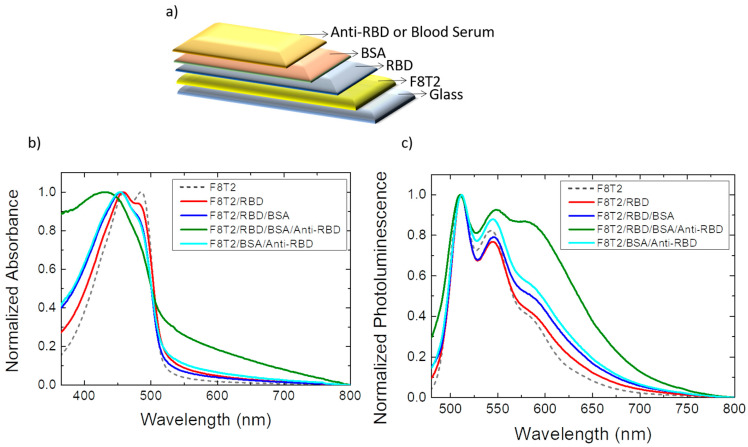
(**a**) Assembly diagram of the optical biosensor configuration GLASS/F8T2/RBD/BSA/anti-RBD. Optical properties of films for construction of the optical biosensor: (**b**) normalized absorption spectrum; (**c**) normalized photoluminescence spectrum (excitation at 450 nm). Absorption and photoluminescence spectra of the F8T2 film were plotted by comparison.

A common problem in biosensors is non-specific adsorption because the non-specific signal can cover the actual detection signal. Therefore, to minimize non-specific interactions of optical biosensors, in the second configuration tested, bovine serum albumin (BSA) was used as a surface blocker after the immobilization of the RBD antigen on F8T2. The surface blocking ensures that the anti-RBD analyte will interact only with the RBD antigen. The utilization of BSA as a blocker to saturate free binding sites on the surface of biosensors has been studied widely and applied in different technologies [29,30,31,32]. This second configuration is depicted in Figure 2a.

The absorption spectra (Figure 2b) show the same appearance as in the previous case. As the film layers were filled, there was an increase in the spectrum in the 350–442 nm region and also in the 510–750 nm region, where the greatest increase was related to the complete biosensor film, that is, with all layers for the study of COVID-19 detection. This study also analyzed the interaction of anti-RBD with F8T2 in the configuration of F8T2/BSA/anti-RBD. In this situation, the absorption spectrum of the film did not show significant changes.

For the photoluminescence spectrum (Figure 2c), it is possible to observe that the spectrum of the film composed of all layers of the biosensor is very different from all other cases (including the study of the interaction of anti-RBD with F8T2 in the F8T2/BSA/anti-RBD configuration). It features 3 well-defined peaks at 510 nm, 550 nm, and 585 nm. Comparing the interaction of the anti-RBD antibody with films with and without RBD, it is clear that the shift in photoluminescence occurred only when the antigen was added to the device. Therefore, the antibody binding to the antigen on the surface induces the photoluminescence emission by F8T2.

**Figure 3:**
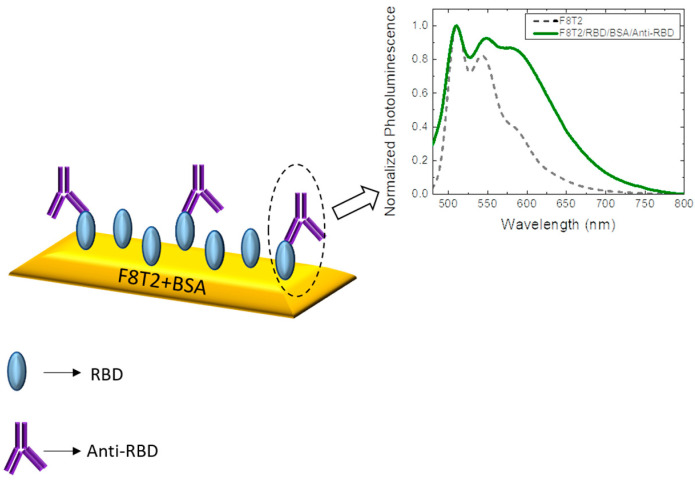
Representation of the RBD/anti-RBD binding generating the alteration of the photoluminescence spectrum.

The antibody-antigen biosensor is a compact analytical device that uses the immunochemical reaction of the RBD antigen with the anti-RBD antibody. Antibodies are complex protein molecules in a “Y” shape. With the help of the BSA surface blocking, the anti-RBD adjusts in a particular way to the RBD antigen, as shown in Figure 3. The deposition of another antigen or antibody under the same tested conditions would imply the non-occurrence of the anti-RBD/RBD antigen binding due to the immunological interaction specificity [9].

When used in biosensors, conjugated polymers convert a biochemical signal resulting from the interaction of a biological component into a measurable signal [18,33,34]. Studies show that the binding of a specific antibody to the antigen can transfer electrons to these materials through redox or enzymatic reactions [35]. In this case, the electron transfer to F8T2, through binding of the RBD antigen to the anti-RBD, generated a more significant emission in the region from 565 nm to 750 nm, leading to an understanding that this structure provides a possible means of detecting the antibody of the infectious disease caused by the new coronavirus via photoluminescence spectroscopy.

In order to study the stability of the optical biosensor, both the sample without BSA and the sample with BSA were subjected to the photoluminescence measurement process after 25 days of manufacture, that is, 25 days after the primary measurement. For this study, samples were stored under ambient temperature and pressure conditions.

**Figure 4:**
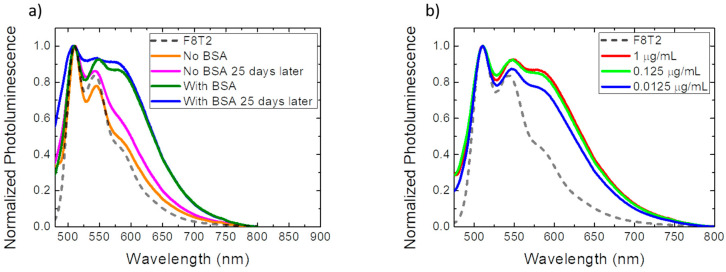
(**a**) Normalized photoluminescence spectrum of the optical biosensor for stability study; (**b**) normalized photoluminescence spectrum of the optical biosensor for the study of the concentration. The photoluminescence spectrum of the film with F8T2 was plotted by comparison.

In this comparison, it was observed, as shown in Figure 4a, that the sample without BSA, despite showing a slight increase in the photoluminescence spectrum as compared to the spectrum carried out on the same day of manufacture, does not exhibit the characteristic of possible antigen/antibody binding due to non-specific connections that occurred without the use of a surface blocker. On the other hand, the sample with BSA presented a more accentuated emission in the region of 510 nm–595 nm when compared to the measurement carried out on the day of manufacture. It also exhibited smoothing at the three characteristic peaks of the primary measure. This phenomenon can be explained by the occurrence of a greater evaporation of water from the film after 25 days. With less water, a decrease in the distance of the antigen/antibody binding may be occurring, facilitating this binding and strengthening its bond, resulting in an intensification of the signal detected by the biosensor [36].

A study of the anti-RBD concentration required to generate a signal in the optical biosensor was also carried out. As shown in Figure 4b, 3 different concentrations were analyzed: 1 µg/mL, 0.125 µg/mL, and 0.0125 µg/mL. When comparing the concentrations of 1 µg/mL and 0.125 µg/mL, the detected signal of the antigen/antibody complex showed almost no variation, and even with a concentration far below (0.0125 µg/mL), the antigen/antibody bond was detected despite the signal-intensity reduction. Given this, the optical biosensor showed high sensitivity even with very low concentrations of antibodies, which is an important contribution to the development of new detectors, aiming for a lower cost and less waste generation.

A preliminary analysis was also performed using blood serum from COVID-19 patients instead of anti-RBD antibodies. This setup is described in Figure 2a, and for this first analysis, 1000× diluted blood sera were used.

**Figure 5:**
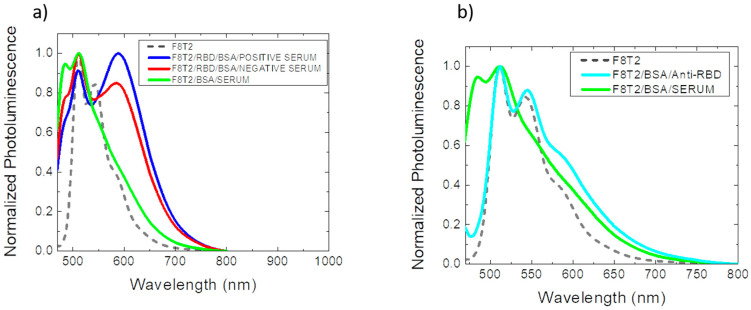
(**a**) Normalized photoluminescence spectrum of the optical biosensor using blood serum for the detection of COVID-19; (**b**) Normalized photoluminescence spectrum of the interaction of anti-RBD with F8T2 and the interaction between blood serum and F8T2.

In this comparison, shown in Figure 5a, it is notable that the interaction of the serum with the whole system behaved differently from the previous case. First, the measurement performed on the film composed of F8T2/BSA/SERUM exhibited a high interaction of the serum with the F8T2 transducer, with a peak at 483 nm in its photoluminescence spectrum. This strong interaction was not observed in the previous experiment, which used purified anti-RBD, as shown in Figure 5b. The purified anti-RBD guaranteed the presence of only the desired antibody in the samples. Therefore, as serum contains several components, such as water, proteins, peptides, electrolytes, organic residues, and a variety of other small molecules [37], these components showed a more significant interaction with F8T2 in this region, making this peak irrelevant for the study of antigen/antibody interaction.

**Figure 6:**
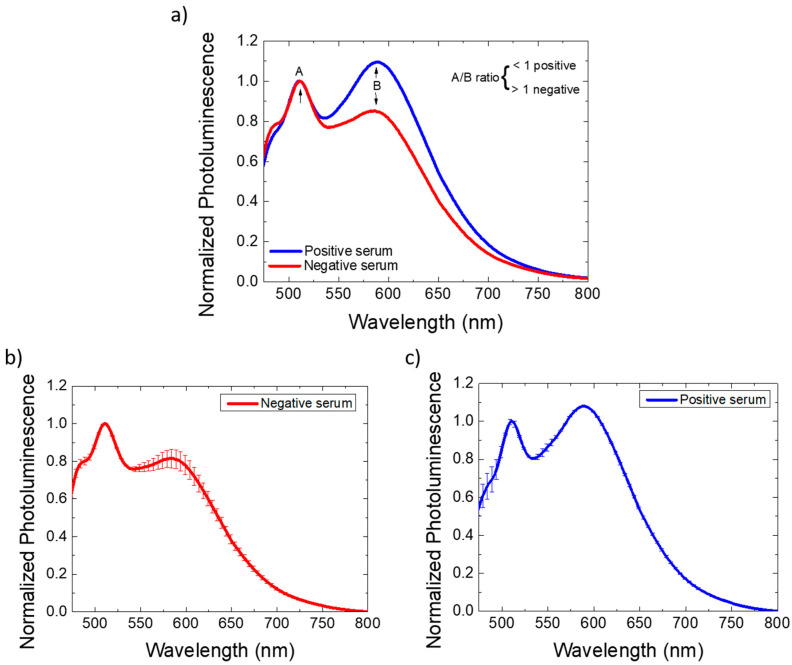
(**a**) Normalized photoluminescence spectrum of the optical biosensor making the peak ratio. The peak ratio between bands A and B (A/B) was <1 to the COVID-19 positive serum, while the negative serum sample had an A/B ratio > 1. Comparison of the photoluminescence spectra of the devices using blood sera diluted 1000 and 3000 times: (**b**) negative sera and (**c**) positive sera.

Despite the serum–transducer background interaction, a difference in the ratio of the 585 nm and 510 nm peaks was measurable for the positive serum. The film with only F8T2 presented a first peak that was more intense than the second or third more discrete peaks. The complete film photoluminescence spectrum using the negative serum, although more extensive, possibly due to the sum of the second and third peaks, kept the proportion of the peaks similar to that observed for F8T2. Adding a positive serum on the biosensor, the proportion of the peaks reversed. Peak inversions may suggest that the interaction of anti-RBD with RBD emits a differential PL signal. As shown in Figure 6a, the peak ratio between bands A and B (A/B) was <1 (0.91) to the COVID-19 positive serum, while the negative serum sample had an A/B ratio > 1 (1.18). The positive sample had a 0.91 A/B ratio, while the negative had an A/B ratio of 1.18.

Finally, for a better comparative analysis, the sensing devices that use negative and positive sera also underwent an evaluation of the influence of their concentration, as shown in Figure 6b,c. For this analysis, the sera were diluted 1000 and 3000 times. It was possible to observe, in addition to the persistence of the peak proportions, the high sensitivity due to the small variation in the photoluminescence spectrum.

## 4. Conclusions

In summary, we have demonstrated the fabrication of a simple optical biosensor using the photoluminescence technique for the diagnosis of COVID-19 through antigen/antibody interaction. In this study, the commercial copolymer F8T2 was used as a signal transducer, the RBD antigen from the spike protein of SARS-CoV-2, BSA as a surface blocker, the purified anti-RBD IgG antibody produced in rabbits, and finally, it was used in place of anti-RBD blood serum from both patients who tested positive and negative for COVID-19. A change in the photoluminescence spectrum was observed when RBD/anti-RBD binding took place. Using blood sera instead of anti-RBD led to greater interaction with F8T2. Still, it was possible to detect COVID-19 through the ratio of the peaks in the photoluminescence spectra. A ratio < 1 could show that the material used tested positive for COVID-19, and a ratio > 1 showed a negative result. Furthermore, it was demonstrated that the optical biosensor showed high sensitivity, good stability, and a short response time, providing an insight into its large-scale application in the detection of other infectious diseases caused by a wide range of viruses.

## Data Availability

The data that support the findings of this study are available from the corresponding authors upon reasonable request.
